# MicroRNA-761 promotes the sensitivity of colorectal cancer cells to 5-Fluorouracil through targeting FOXM1

**DOI:** 10.18632/oncotarget.20109

**Published:** 2017-08-10

**Authors:** Shuguang Cao, Limiao Lin, Xuanping Xia, Hao Wu

**Affiliations:** ^1^ Department of Gastroenterology, The Second Affiliated Hospital and Yuying Children’s Hospital of Wenzhou Medical University, Wenzhou 325000, Zhejiang, China

**Keywords:** colorectal cancer, miR-761, 5-Fluorouracil, FOXM1

## Abstract

Resistance to chemotherapy is a big challenge for treatment of patients with colorectal cancer; however; the mechanism underlying chemoresistance in colorectal cancer cell has not been elucidated. MicroRNAs (miRNAs) are new players in the development of drug chemoresistance. In our study, we indicated that overexpression of miR-761 promoted the sensitivity of colorectal cancer cells to 5-Fluorouracil (5-FU). miR-761 expression was downregulated in colorectal cancer cell lines and tissues. miR-761 expression was lower in patients with low grade than in patients with high grade. In additon, we showed that elevated expression of miR-761 suppressed colorectal cancer cell proliferation, cell cycle, colony formation and cell invasion. We identified that FOXM1 was a direct target gene of miR-761 in colorectal cancer cell. FOXM1 expression was upregulated in colorectal cancer tissues compare to the adjacent non-tumor tissues. MiR-761 expression was negatively associated with the expression of FOXM1 in colorectal cancer tissues. Elevated expression of FOXM1 suppressed the sensitivity of miR-761-overexpressing HT29 cells to 5-FU. We also indicated that FOXM1 overexpression promoted cell proliferation, cycle and invasion of miR-761-overexpressing HT29 cells. These data suggested that miR-761 played a tumor suppressor miRNA in colorectal cancer progression and reduced miR-761 expression might be a major mechanism for 5-FU resistance in colorectal cancer cell.

## INTRODUCTION

Colorectal cancer (CRC) accounts for about 13% of all tumors and the 2th leading cause of tumor-related death in developed countries [1–5]. Despite therapeutic strategies such as chemotherapy, surgery and radiotherapy that have been applied in CRC treatment, the prognosis of the colorectal cancer patients has not been satisfied over the last 20 years [6–9]. The molecular mechanism and pathological process of the colorectal cancer were complicated and was still not fully understood [10–12]. Therefore, it is important to elucidate the molecular mechanisms of colorectal cancer to find the new therapeutic strategies.

MicroRNAs (miRNAs) are small, evolutionarily conserved and short non-coding RNAs that work as posttranscriptional gene governor through targeting to the partially complementary sequences in 3′UTR of target mRNAs [13–17]. Numbers of studies have demonstrated that miRNAs play pivotal roles in diverse biological processes such as cell proliferation, differentiation, metastasis, angiogenesis, and invasion [18–22]. Increasing evidence demonstrated that miRNAs were dysregulated in many tumors including osteosarcoma, gastric cancer, hepatocellular carcinoma, lung cancer, gallbladder carcinoma and bladder cancer [23–28]. Recently, a lot of studies have demonstrated that miRNAs are a new player in the development of drug chemoresistance [29–31]. MiRNAs are deregulated expression in the chemoresistant and chemosensitive cells [32, 33].

Previous studies suggested that miR-761 acted important roles in the development and progression of various tumors [34–37]. However, the role of miR-761 in the colorectal cancer is still uncovered. In our study, we demonstrated that overexpression of miR-761 promoted the sensitivity of colorectal cancer cells to 5-FU. We demonstrated that the expression level of miR-761 was downregulated in colorectal cancer tissues and cell lines. We also indicated that elevated expression of miR-761 suppressed colorectal cancer cell proliferation, cell cycle, colony formation and cell invasion.

## RESULTS

### miR-761 promoted the sensitivity of colorectal cancer cells to 5-FU

**Figure 1:**
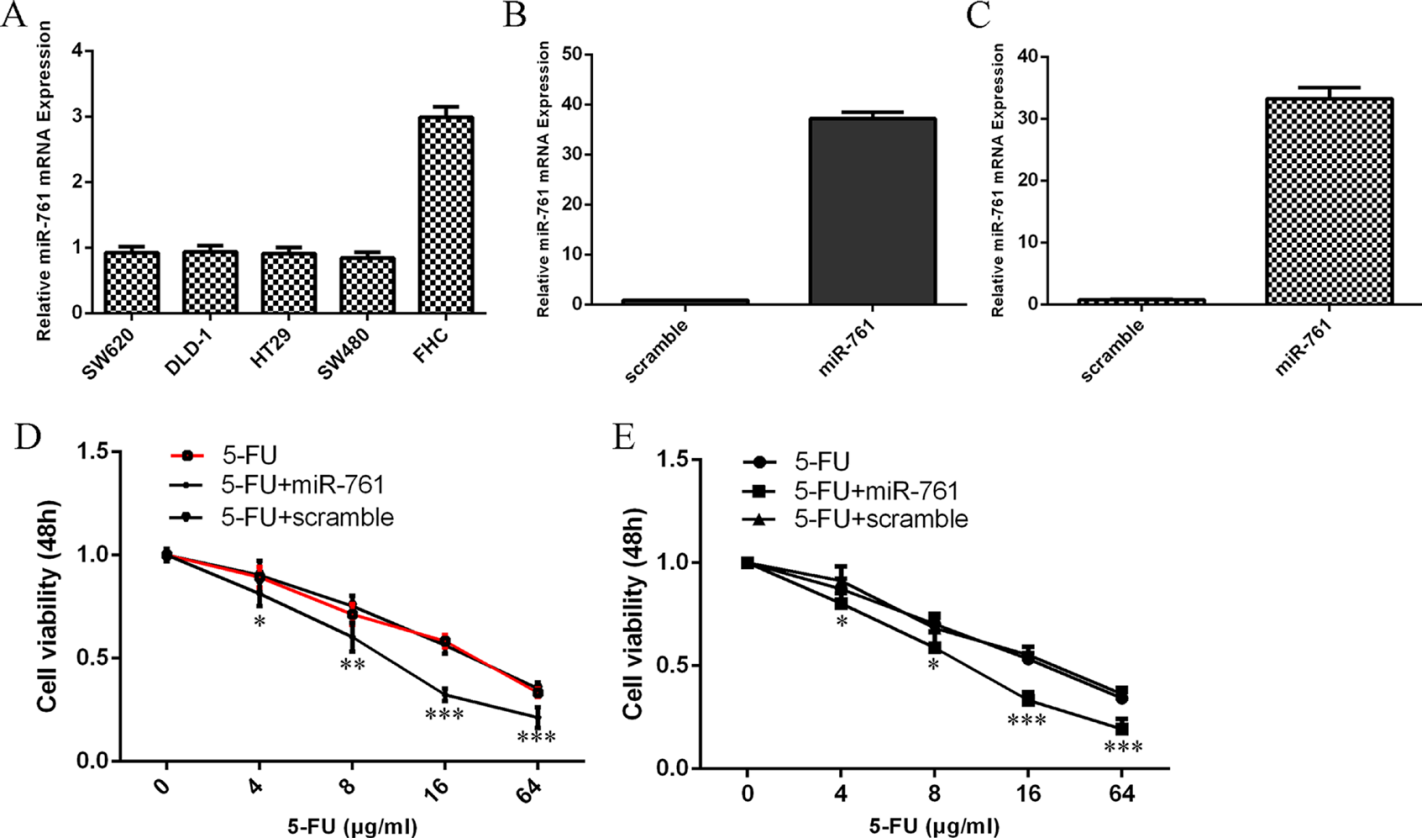
miR-761 promoted the sensitivity of colorectal cancer cells to 5-FU (**A**) The miR-761 expression in the colorectal cancer cell (HT29, SW480, SW620 and DLD-1) and normal colon epithelium cell line (FHC) was detected by qRT-PCR analysis. U6 was used as the internal control. (**B**) The expression level of miR-761 in the HT29 cell treated with miR-761 mimic was measured by qRT-PCR analysis. (**C**) The expression level of SW480 in the HT29 cell treated with miR-761 mimic was measured by qRT-PCR analysis. (**D**) The response of HT29 cells to 5-FU was increased after treated with the miR-761 mimic compared to the scramble-transfected cells. (**E**) The response of SW480 cells to 5-FU was increased after treated with the miR-761 mimic compared to the scramble-transfected cells. ^*^p < 0.05; ^**^p < 0.01 and ^***^p < 0.001.

The expression level of miR-761 was downregulated in colorectal cancer cells (HT29, SW480, SW620 and DLD-1) compared to normal colon epithelium cell line (FHC) (Figure 1A). qRT-PCR analysis indicated that miR-761 mimic prommoted miR-761 expression both in the HT29 and SW480 cell (Figure 1B and 1C). The response of HT29 and SW480 cells to 5-FU was increased after treated with miR-761 mimic compared to the scramble-transfected cells (Figure 1D and 1E).

### miR-761 expression was downregulated in colorectal cancer tissues

**Figure 2:**
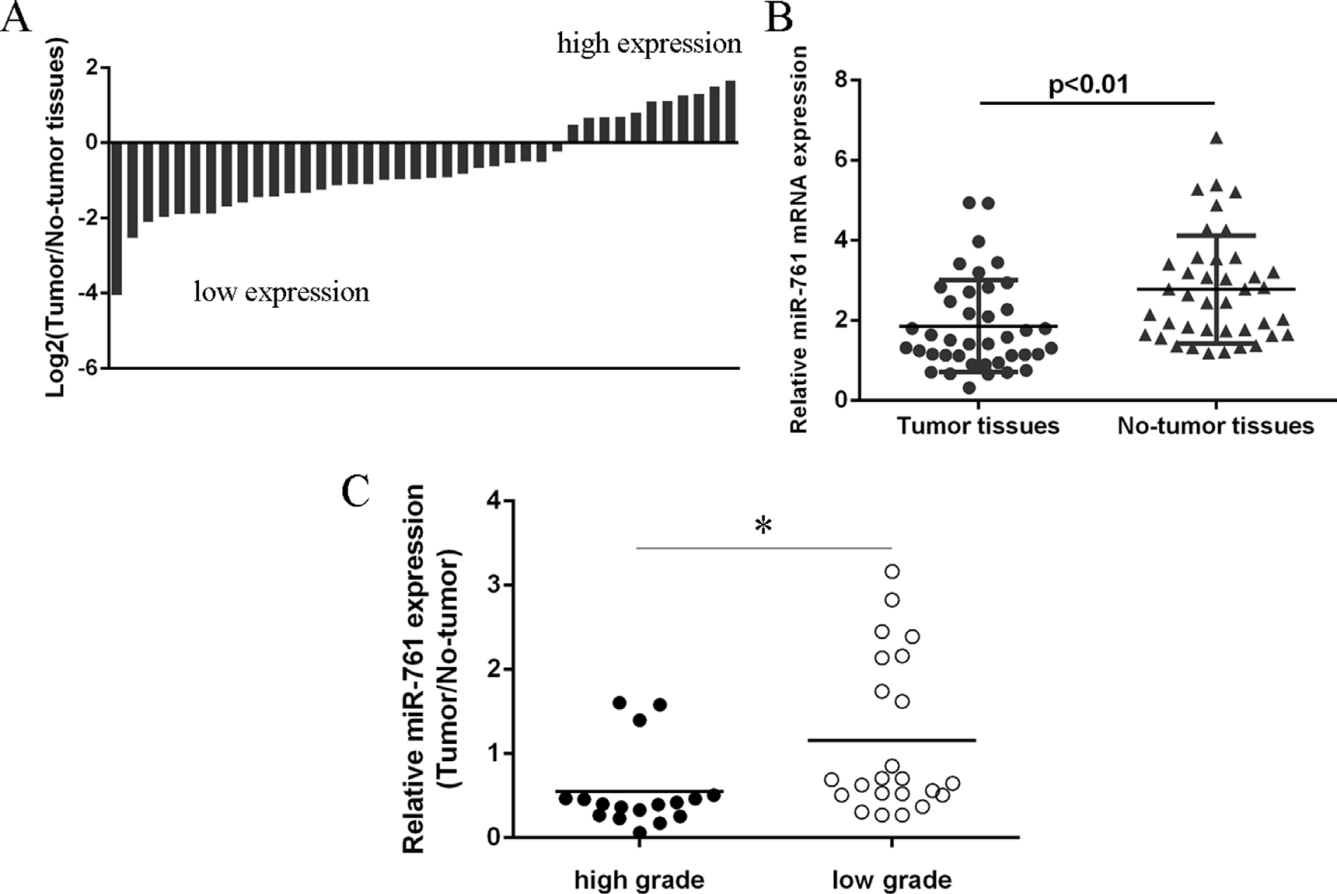
miR-761 expression was downregulated in colorectal cancer tissues (**A**) The miR-761 expression in the colorectal cancer tissues and the adjacent non-tumor tissues was determined by qRT-PCR. U6 was used as the internal control. (**B**) The expression of miR-761 was lower in the colorectal cancer samples compare to the non-tumor samples. (**C**) The miR-761 expression was lower in the colorectal cancer patients with low grade than in the patients with low grade. ^*^p < 0.05.

We then measured the miR-761 expression in the colorectal cancer tissues. Our data showed that miR-761 expression was downregulated in 29 colorectal cancer patients compare to the adjacent non-tumor tissues (Figure 2A). The expression of miR-761 was lower in colorectal cancer samples compare to the non-tumor samples (Figure 2B). In addition, miR-761 expression was lower in patients with low grade than in patients with high grade (Figure 2C).

### Elevated expression of miR-761 suppressed colorectal cancer cell proliferation

**Figure 3:**
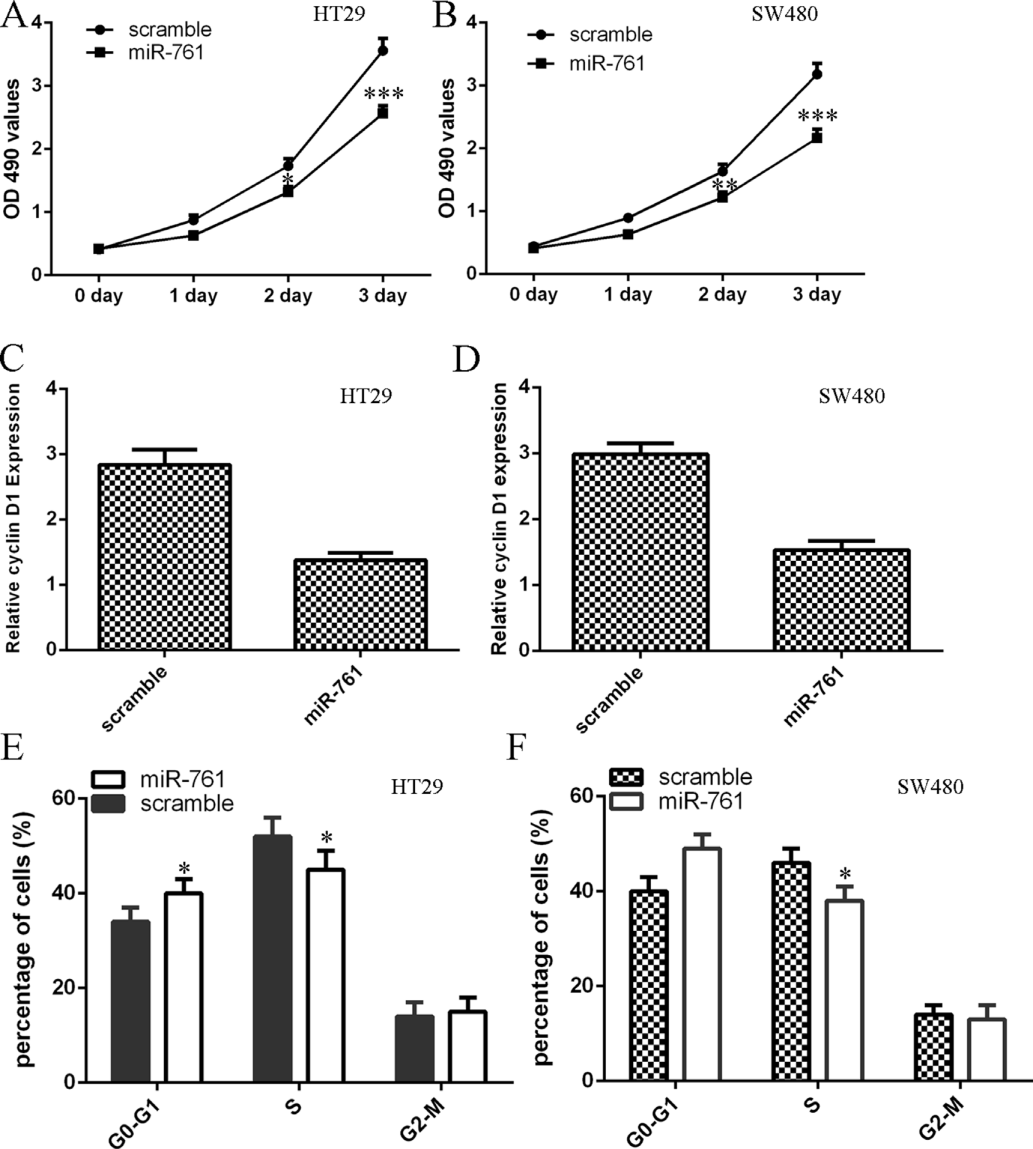
Elevated expression of miR-761 suppressed colorectal cancer cell proliferation (**A**) Overexpression of miR-761 suppressed the HT29 cell proliferation. (**B**) Ectopic expression of miR-761 suppressed the SW480 cell proliferation. (**C**) Elevated expression of miR-761 decreased the cyclin D1 expression in the HT29 cell. (**D**) Overexpression of miR-761 decreased the cyclin D1 expression in the SW480 cell. (**E**) Ectopic expression of miR-761 decreased the HT29 cell cycle. (**F**) Elevated expression of miR-761 suppressed the SW480 cell cycle. ^*^p < 0.05; ^**^p < 0.01 and ^***^p < 0.001.

We demonstrated that overexpression of miR-761 decreased cell proliferation in colorectal cancer cell lines HT29 and SW480 (Figure 3A and 3B). In addition, ectopic expression of miR-761 inhibited cyclin D1 expression in both HT29 and SW480 cell (Figure 3C and 3D). Moreover, elevated expression of miR-761 decreased HT29 and SW480 cell cycle (Figure 3E and 3F).

### Overexpression of miR-761 decreased colorectal cancer cell colony formation and invasion

**Figure 4:**
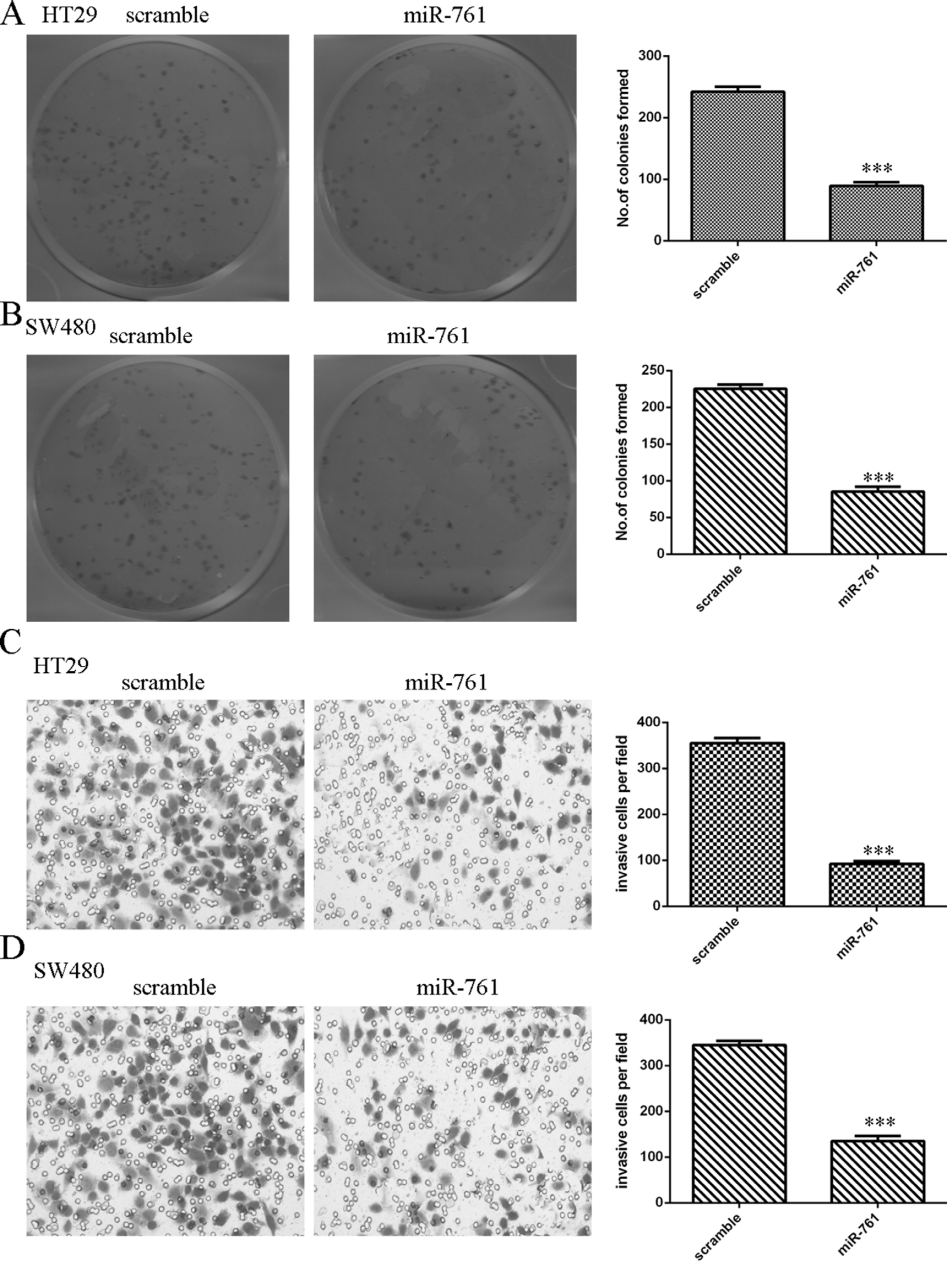
Overexpression of miR-761 decreased the colorectal cancer cell colony formation and invasion (**A**) Ectopic expression of miR-761 suppressed the HT29 cell colony formation. The relative colony formation numbers were shown in the right. (**B**) Overexpression of miR-761 suppressed the SW480 cell colony formation. The relative colony formation numbers were shown in the right. (**C**) miR-761 overexpression inhibited the cell invasion in the HT29 cell. The relative invasive cells were shown. (**D**) Elevated expression of miR-761 suppressed the SW480 cell invasion. The relative invasive cells were shown. ^***^p < 0.001.

Ectopic expression of miR-761 suppressed HT29 and SW480 cell colony formation (Figure 4A and 4B). Moreover, we performed the invasion assay to measure cell invasion ability. Our data indicated that miR-761 overexpression decreased the HT29 and SW480 cell invasion (Figure 4C and 4D).

### FOXM1 was a direct target gene of miR-761

**Figure 5:**
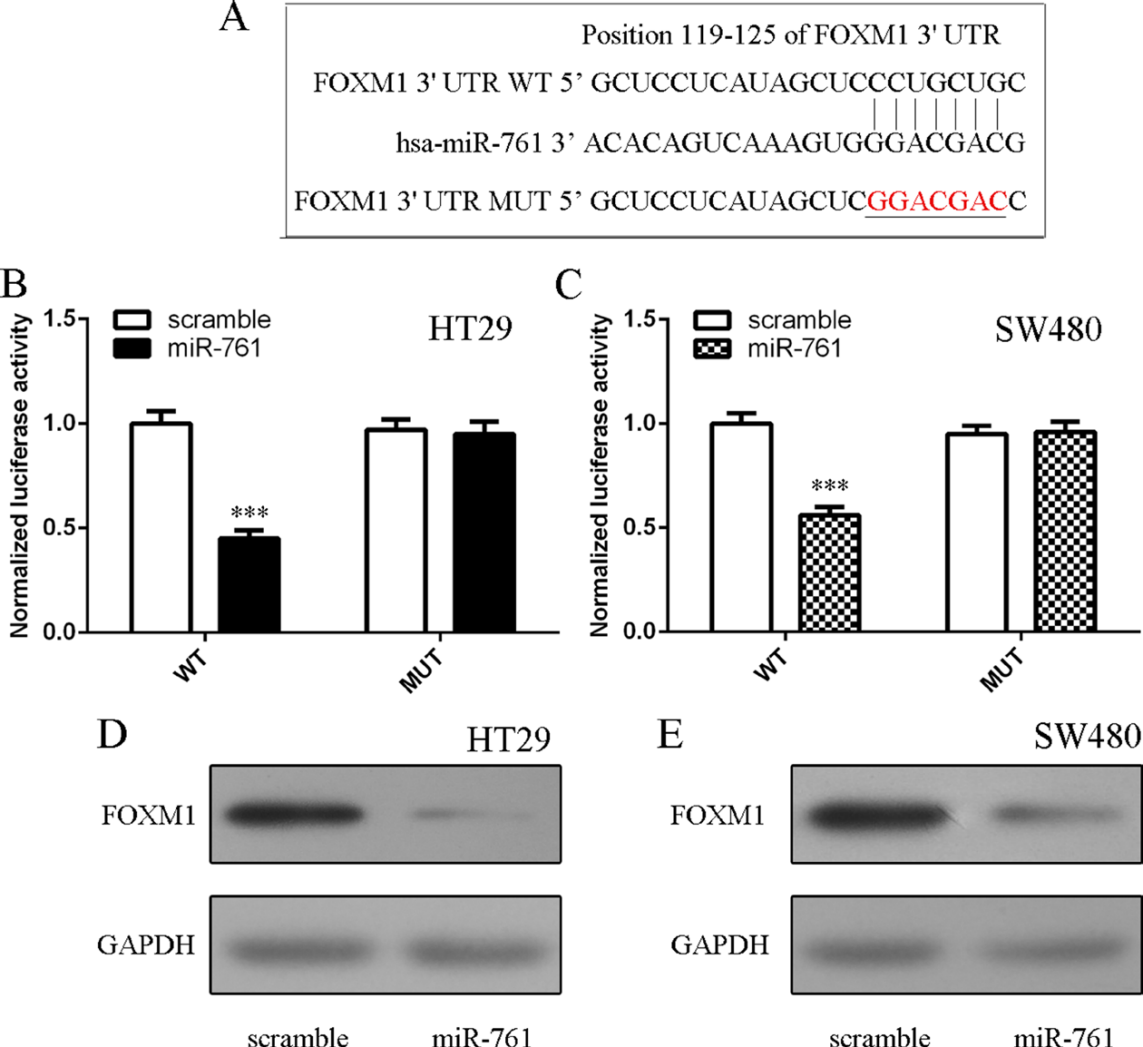
FOXM1 was a direct target gene of miR-761 (**A**) The putative binding sites of miR-761 and FOXM1 are shown. (**B**) Ectopic expression of miR-761 decreased the luciferase activity of wild-type 3′UTR of the FOXM1 construct, but not in the mutated-type 3′UTR of the FOXM1 vector in the HT29 cell. (**C**) Elevated expression of miR-761 decreased the luciferase activity of wild-type 3′UTR of the FOXM1 construct, but not in the mutated-type 3′UTR of the FOXM1 vector in the SW480 cell. (**D**) The protein expression of FOXM1 was measured by Western blot. GAPDH was used the internal control. (**E**) Elevated expression of miR-761 inhibited the protein expression of FOXM1 both in the SW480 cell. ^***^p < 0.001.

To study the molecular mechanism of miR-761 in colorectal cancer cell, we used the website TargetScan database to identify potential target gene of miR-761. The putative binding site of miR-761 and FOXM1 was shownAs shown in Figure 5A. Moreover, elevated expression of miR-761 suppressed luciferase activity of wild-type 3′UTR of the FOXM1 construct, but not in the mutated-type 3′UTR of the FOXM1 vector in HT29 and SW480 cells (Figure 5B and 5C). Furthermore, elevated expression of miR-761 inhibited the protein expression of FOXM1 in HT29 and SW480 cells (Figure 5D and 5E).

### FOXM1 expression was upregulated in colorectal cancer tissues

**Figure 6:**
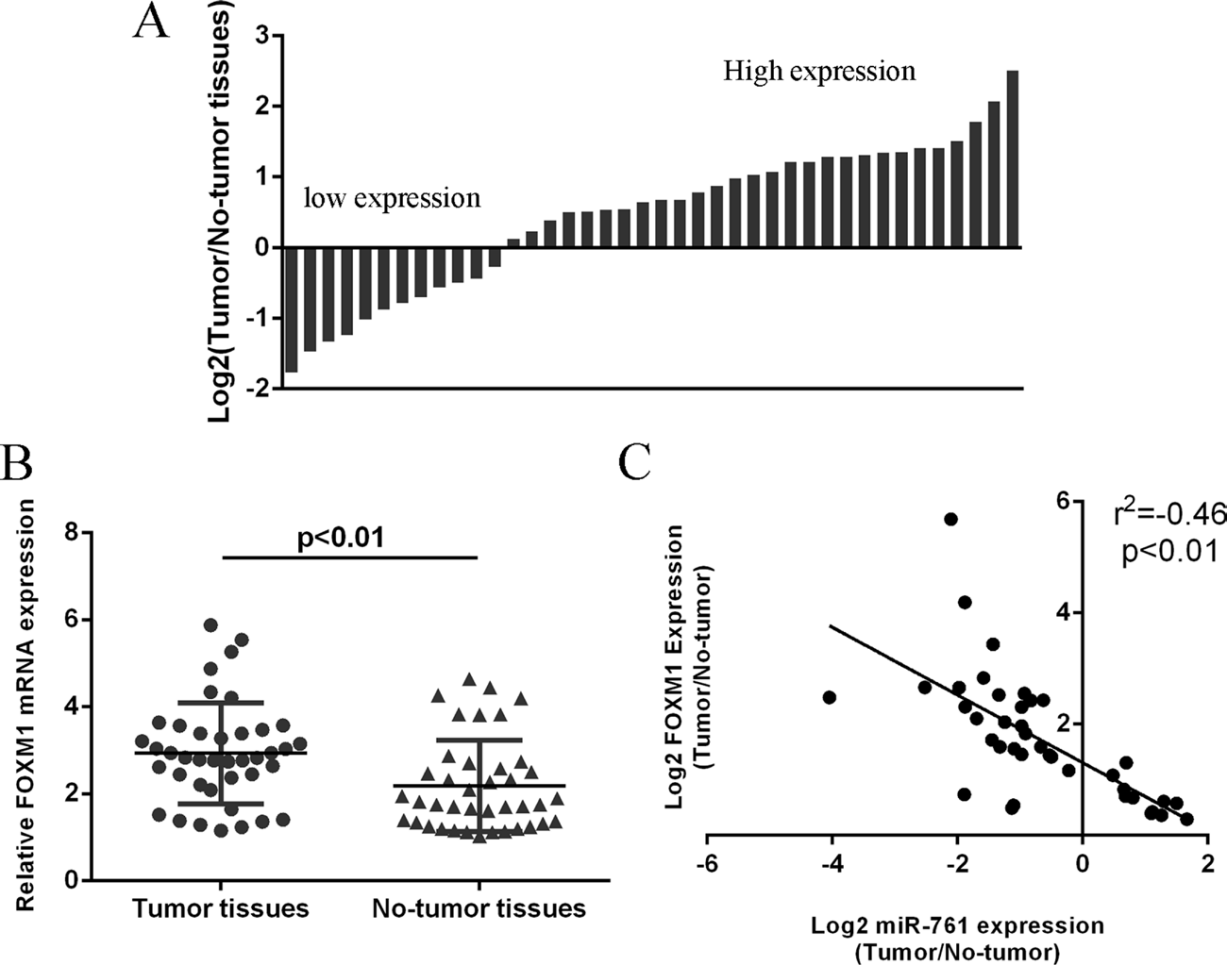
FOXM1 expression was upregulated in colorectal cancer tissues (**A**) The FOXM1 expression in the colorectal cancer tissues and adjacent non-tumor tissues was measured by qRT-PCR and U6 was used as the internal control. (**B**) The expression of FOXM1 was higher in the colorectal cancer samples than in the non-tumor samples. (**C**) The miR-761 expression was negatively associated with the expression of FOXM1 in the colorectal cancer tissues.

We next detected FOXM1 expression in colorectal cancer tissues. Our data showed that FOXM1 expression was upregulated in 28 colorectal cancer patients compared to the adjacent non-tumor tissues (Figure 6A). The expression of FOXM1 was higher in colorectal cancer samples compare to the non-tumor samples (Figure 6B). In addition, miR-761 expression was negatively associated with the expression of FOXM1 in colorectal cancer tissues. (Figure 6C).

### miR-761 promoted the sensitivity of colorectal cancer cells to 5-FU through targeting FOXM1

**Figure 7:**
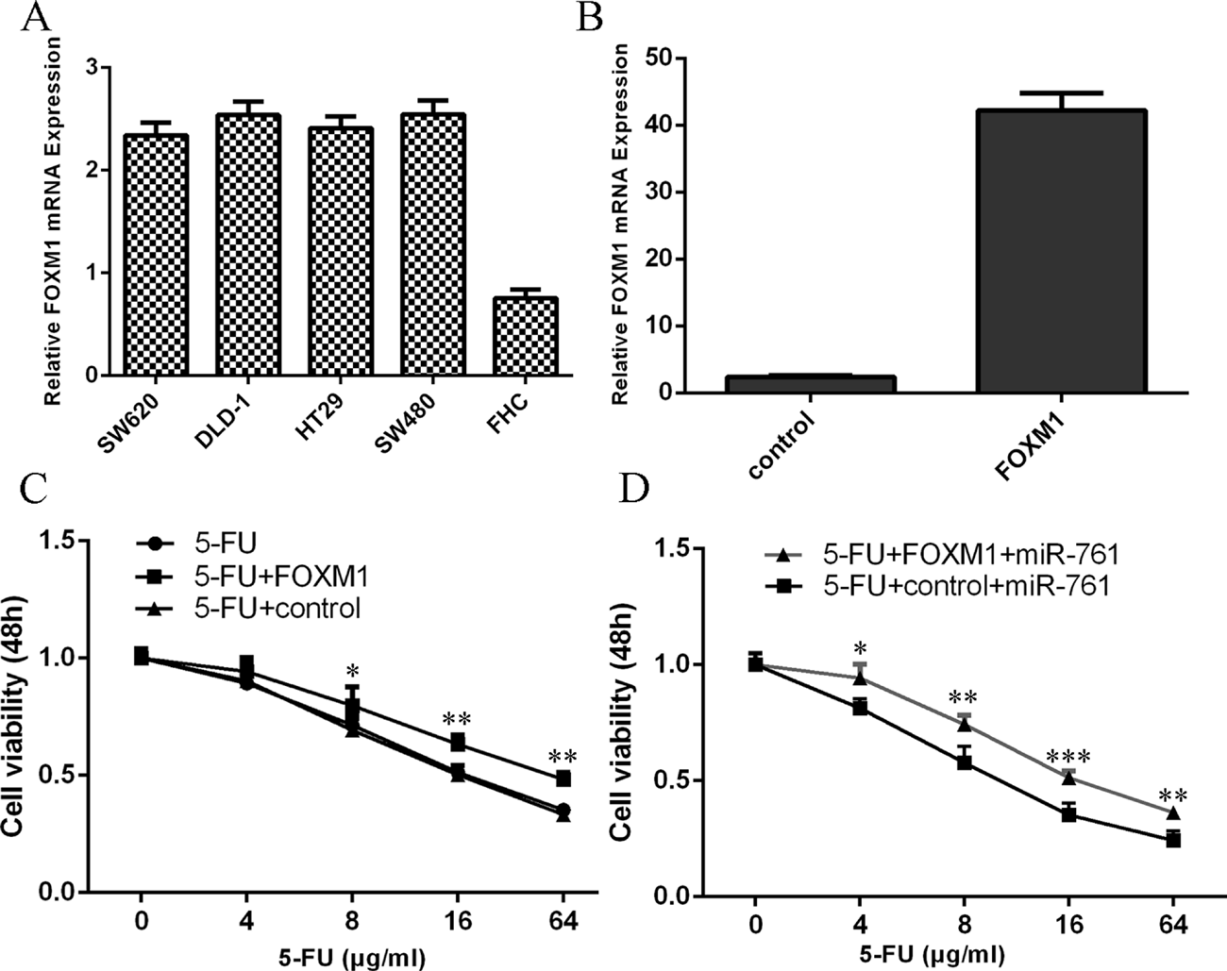
miR-761 promoted the sensitivity of colorectal cancer cells to 5-FU through targeting FOXM1 (**A**) The expression level of FOXM1 in the olorectal cancer cell (HT29, SW480, SW620 and DLD-1) and normal colon epithelium cell line (FHC) was measured by qRT-PCR. (**B**) The expression of FOXM1 in the HT29 cell with FOXM1 vector was determined by qRT-PCR. (**C**) The response of HT29 cell to 5-FU was enhanced after treated with the FOXM1 vector compared with the cotrol-transfected cells. (**D**) The response of miR-761-overexpressing HT29 cell to 5-FU was rescued after transfection with the FOXM1 construct compared to the control vector. ^*^p < 0.05; ^**^p < 0.01 and ^***^p < 0.001.

The expression level of FOXM1 was upregulated in the olorectal cancer cell (HT29, SW480, SW620 and DLD-1) compared to normal colon epithelium cell line (FHC) (Figure 7A). The expression of FOXM1 was upregulated in the HT29 cell after tranfected with FOXM1 vector (Figure 7B). The response of HT29 cell to 5-FU was enhanced after treated with FOXM1 vector compared with the cotrol-transfected cells (Figure 7C). Moreover, the response of miR-761-overexpressing HT29 cell to 5-FU was rescued after transfection with the FOXM1 construct compared to the control vector (Figure 7D).

### miR-761 suppressed colorectal cancer cell proliferation and invasion by downregulating FOXM1

**Figure 8:**
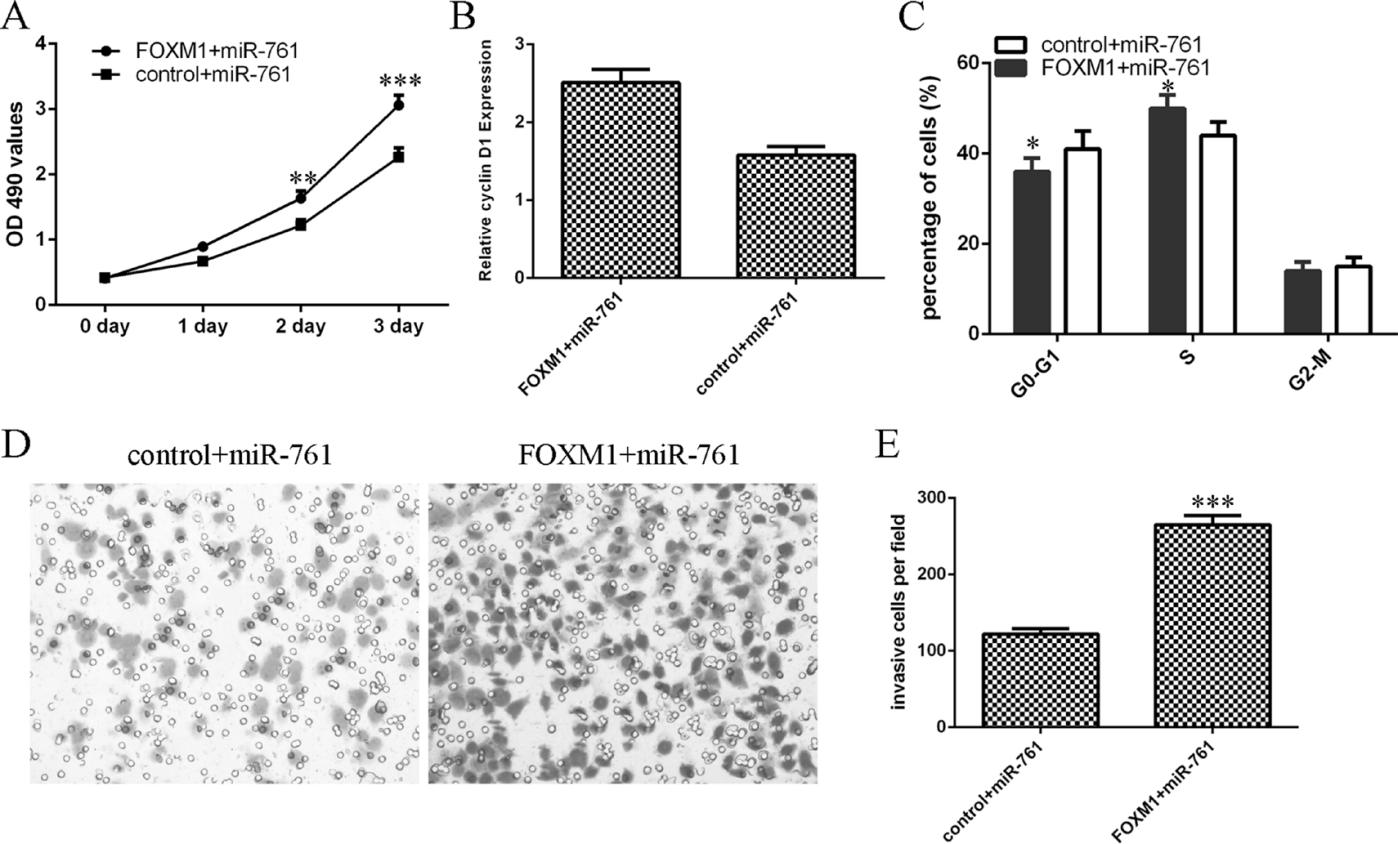
miR-761 suppressed the colorectal cancer cell proliferation and invasion by downregulating FOXM1 (**A**) Overexpression of FOXM1 rescued miR-761-overexpressing HT29 cell proliferation. (**B**) The mRNA expression of FOXM1 was measured by qRT-PCR. GAPDH was used as the internal control. (**C**) Elevated expression of FOXM1 promoted the miR-761-overexpressing HT29 cell cycle. (**D**) miR-761 overexpression increased the miR-761-overexpressing HT29 cell invasion. (**E**) The relative invasive cells were shown. ^*^p < 0.05; ^**^p < 0.01 and ^***^p < 0.001.

Overexpression of FOXM1 rescued miR-761-overexpressing HT29 cell proliferation (Figure 8A). In addition, elvelated expression of FOXM1 enhanced cyclin D1 expression in the miR-761-overexpressing HT29 cell (Figure 8B). Elevated expression of FOXM1 promoted the miR-761-overexpressing HT29 cell cycle (Figure 8C). Furthermore, miR-761 overexpression increased the miR-761-overexpressing HT29 cell invasion (Figure 8D and 8E).

## DISCUSSION

Chemoresistance is a great challenge for treating patients with advanced colorectal cancer [30, 31]. It is important for us to find new strategies for conquering the resistance to chemotherapeutic events. In this study, we indicated that overexpression of miR-761 promoted the sensitivity of colorectal cancer cells to 5-FU. We demonstrated that the expression level of miR-761 was downregulated in colorectal cancer cell (HT29, SW480, SW620 and DLD-1) compared to normal colon epithelium cell line (FHC). Furthermore, we showed that the expression of miR-761 was downregulated in colorectal cancer tissues. miR-761 expression was lower in the patients with low grade than in patients with high grade. In additon, we indicated that elevated expression of miR-761 suppressed colorectal cancer cell proliferation, cell cycle, colony formation and cell invasion. We identified that FOXM1 was a direct target gene of miR-761 in colorectal cancer cell. FOXM1 expression was upregulated in colorectal cancer tissues compare to the adjacent non-tumor tissues. miR-761 expression was negatively associated with the expression of FOXM1 in colorectal cancer tissues. Elevated expression of FOXM1 suppressed the sensitivity of miR-761-overexpressing HT29 cells to 5-FU. We also indicated that FOXM1 overexpression promote cell proliferation, cycle and invasion of miR-761-overexpressing HT29 cells. These data suggested that miR-761 played a tumor suppressor miRNA in colorectal cancer progression and reduced miR-761 expression may be a major mechanism for 5-FU resistance in colorectal cancer cell.

Previous studies suggested that miR-761 acted important roles in the development and progression of various tumors [34–37]. Yan et al [34]. indicated that miR-761 expression was upregulated in non-small cell lung cancer (NSCLC) serum and tissues and overexpression of miR-761 increased NSCLC cell proliferation and metastasis through regulating ING4 and TIMP2 expression. Zhou et al [35]. found that miR-761 expression was upregulated in hepatocellular carcinoma tissues and inhibted expression of miR-761 impaired mitochondrial function and inhibited tumor metastasis and growth through upregulating Mitofusin-2. Guo et al [37]. demonstrated that miR-761 expression was increased in breast tumor tissues and cell lines and overexpression of miR-761 promoted cell proliferation, colony formation, invasion and migration through targeting tripartite motif-containing 29 (TRIM29). However, Shi et al [36]. demonstrated that the expression of miR-761 was downregulated in ovarian cancer tissues and miR-761 overexpression decreased ovarian cancer cell invasion and proliferation through inhibiting MSI1 expression. Until now, there are no references reported about the role of miR-761 in the development of colorectal cancer. In our study, we indicated that miR-761 expression was downregulated in colorectal cancer tissues. miR-761 expression was lower in patients with low grade than in patients with high grade CRC. Moreover, we indicated that elevated expression of miR-761 suppressed colorectal cancer cell proliferation, cell cycle, colony formation and cell invasion. Theses data suggested that miR-761 played as a tumor suppressor miRNA in colorectal cancer progression.

Forkhead box M1 (FOXM1) is a key transcription factor characterized by a winged-helix DNA-binding domain, which is also known as HNF-3/fork-head homolog 11, forkheaddrosophila homolog like 16 and Trident and membrane palmitoylated protein 2 [38–40]. Increasing studies showed that FOXM1 played critical roles in cell cycle, DNA replication, invasion, migration, angiogenesis and drug resistance [41–44]. FOXM1 expression was found to be upregulated in several tumors such as gastric cancer, hepatocellular carcinoma, lung caner, glioma and also colorectal cancer [42, 45–48]. For example, Zheng et al [49]. demonstrated that overexpression of FOXM1 promoted colorectal cancer cell invasion and migration through regulating pituitary tumor transforming gene (PTTG1) expression. Zhang et al [50]. also found that FOXM1 expression was upregulated in colorectal cancer tissues and silencing of FOXM1 decreased colorectal cancer cell migration and invasion. Recently, Xie et al. [41]. showed that the expression of FOXM1 was upregulated in the 5-FU nonresponsive CRC patients and inhibition of FOXM1 resensitized resistant CRC cells to 5-FU treatment through regulating ABCC10 expression. In addition, Liu et al. [51]. showed that miR-149 promoted the sensitivity of colorectal cancer cell to 5-FU through targeting FOXM1. In this study, we also found that the expression of FOXM1 was upregulated in colorectal cancer cell lines and tissues. Overexpression of miR-761 suppressed luciferase activity of wild-type 3′UTR of the FOXM1 construct, but not in the mutated-type 3′UTR of the FOXM1 vector. Ectopic expression of miR-761 suppressed the FOXM1 expression. Furthermore, the miR-761 expression was negatively associated with the expression of FOXM1 in colorectal cancer tissues. In addition, miR-761 overexpression promoted the sensitivity of colorectal cancer cells to 5-FU through targeting FOXM1. Ectopic expression of miR-761 inhibited colorectal cancer cell proliferation and invasion by downregulating FOXM1 expression.

In summary, our data suggested that miR-761 was downregualted in colorectal cancer tissues and cells. Overexpression of miR-761 promoted the sensitivity of colorectal cancer cells to 5-FU and ectopic expression of miR-761 suppressed colorectal cancer cell proliferation, cell cycle, colony formation and invasion partly through regulating FOXM1 expression.

## MATERIALS AND METHODS

### Tissue samples and cell culture and transfection

The colorectal tumor tissues and adjacent normal samples utilized in our study were collected from our department. Informed consents were obtained from each patients and this experiment was approved by the review board of The Second Affiliated Hospital and Yuying Children’s Hospital of Wenzhou Medical University. Four colorectal tumor cell lines (HT29, SW480, SW620 and DLD-1) and one normal colon epithelium cell line (FHC) were purchased from the ATCC (MD, USA) and were keplt in the Dulbecco’s modified Eagle’s medium (DMEM). miR-761 and scramble, pcDNA-FOXM1 and control vector were synthesized from the GenePharma Company (Shanghai, China). Colorectal tumor cells were transfected with plasmid by using the Lipofectamin 2000 (Invitrogen, CA, USA) following to the manufacturer’s information.

### RNA extraction and qRT-PCR

Total RNA was extracted from cell or sample using the Trizol reagent (Invitrogen, CA, USA). Quantitative RT-PCR (qRT-PCR) was conducted in the Bio-Rad (Hercules, USA) IQ5 PCR system following to the manufacturer’s instruction to measure the miR-761 and FOXM1 expression. The qPCR reaction was done with the following: 15 minutes at 50°C, 10 minutes at 95°C, followed by 42 cycles at the 95°C for 15 second and 60°C for 1 minute. U6 and GAPDH were conducted as the internal control for miR-761 and FOXM1 respectively. The sequences are listed as follows: miR-761 forward 5′-ACAGCAGGCACAGAC-3′ and reverse 5′-GAGCAGGCTGGAGAA-3′; FOXM1 forward 5′-GTGAATGGTCCAGAAGGAGAC-3′ and reverse 5′-ACCACTTTCCCTACTTTAAGCAC-3′; U6 forward 5′-CTCGCTTCGGCAGCACATATACT-3′ and reverse 5′- ACGCTTCACGAATTTGCGTGTC-3′; and GAPDH were forward 5′- TGTTCGACAGTCAGCCGC-3′ and reverse 5′-GGTGTCTGAGCGATGTGGC-3′.

### Western blot

Western blot assay was done to measure the protein expression of FOXM1. The protein lysate was extracted from cells or samples using the Protein Extraction Reagent (Thermo Fisher Scientific, USA). The protei lysate was separated by 10% SDS-PAGE gel and transfermed to PVDF membrane (Millipore). The membranes were blocked with 5% non-fat milk and then incubated with the fisrt antibodies (FOXM1 and GAPDH, 1:1000 dilution, Abcam). GAPDH was performed as the internal control for candidate genes.

### Luciferase reporter assay

The HT29 and SW480 cell were co-transfected with the pGL-3 FOXM1 reporter construct, pGL-3 control vector and miR-761 or the scramble vector using the Lipofectamin 2000 (Invitrogen, CA, USA) according to the manufacturer’s information. The cell was harvested 24 hours post-transfection and the luciferase reporter assay was measured by the Dual Luciferase Assay (Promega, WI, USA) following to the manufacturer’s information.

### Cell proliferation, invasion and colony formation assays

Cell proliferation was determined using the 3′ (4, 5-dimethylthazol-2-yl)-2, 5-diphenyltetrazolium bromide (MTT) analysis. The cells were cultured into 96 well plates and cell viability was measured the MTT assay according to manufacturer’s information. The absorbance at the 490 nm was read on the spectrophotometer. For cell invasion assay, cell was placed on the upper chamber with Matrigel coated membrane which was added to the serum-free culture medium. In the lower chamber, 10% FBS (fetal bovine serum) was added. After incubation for 24 hours, cell that invased to the bottom of the chamber was fixed with methanol and stained with crystal violet. For cell colony formation assay, the cell was cultured on the 6-well plate and continuted to culture for 2 weeks. The colony cell was then stained with the crystal violet and counted.

### Statistical analysis

The statistical analysis in our study was measured using the SPSS 17.0 software. The numerical data was shown as mean ± SD (standard deviation). The difference between more than two groups was performed with the one-way ANOVA. Student’s *t*-test was used to assess the difference between two groups. *P* < 0.05 was considered as significant.
